# Interpretable Machine Learning Identifies Key Inflammatory and Morphological Drivers of Intracranial Aneurysm Rupture Risk

**DOI:** 10.3390/bioengineering13020226

**Published:** 2026-02-15

**Authors:** Epameinondas Ntzanis, Nikolaos Papandrianos, Petros Zampakis, Vasilios Panagiotopoulos, Constantinos Koutsojannis, Christina Kalogeropoulou, Elpiniki I. Papageorgiou

**Affiliations:** 1 Department of Radiology, University of Patras, 26504 Patras, Greece; e.ntzanis@upatras.gr (E.N.); ckrat@upatras.gr (C.K.); 2 Department of Energy Systems, School of Technology, University of Thessaly, Gaiopolis Campus, 41500 Larisa, Greece; npapandrianos@uth.gr; 3 Department of Interventional Neuroradiology/Endovascular Neurosurgery, University Hospital of Patras, 26500 Patras, Greece; pzampakis@gmail.gr; 4 Department of Neurosurgery, University Hospital of Patras, 26500 Patras, Greece; baspan@upatras.gr; 5 Laboratory of Health Physics & Computational Intelligence, Department of Physiotherapy, School of Rehabilitations Sciences, University of Patras, 26500 Patras, Greece; ckoutsog@upatras.gr

**Keywords:** intracranial aneurysm, rupture risk, explainable AI, Random Forest, LIME, CRP, complement C3, complement C4, neck width, irregular shape

## Abstract

Traditional statistical approaches identify group-level associations between biomarkers and rupture status in intracranial aneurysms (IAs) but often miss nonlinear interactions at the patient level. Methods: The authors retrospectively analyzed 35 saccular IAs in 35 patients (57.1% ruptured) from a single center (2021–2023). Demographics, detailed morphology (e.g., neck width, aspect ratio, VERTI, irregular shape), and multi-site inflammatory/immune markers (CRP; complement C3/C4; IgA/IgG/IgM) were included. After preprocessing (min–max scaling; one-hot encoding), five algorithms (DT, AdaBoost, GBM, XGBoost, RF) were evaluated with stratified five-fold CV and class balancing via random oversampling. The primary model (Random Forest) was tuned with Optuna and explained using global feature importance and LIME. The results showed that baseline RF achieved CV ROC-AUC 0.81 and test ROC-AUC 0.92 (test accuracy 0.857). The tuned RF (with oversampling and Optuna) yielded a mean CV accuracy of 0.85 ± 0.09 and CV ROC-AUC of 0.98 ± 0.07 while maintaining test ROC-AUC of 0.92. The average precision on the test PR curve was 0.97. The most influential predictors combined inflammatory markers (CRP, C3, C4) with morphology (neck width, irregular shape). LIME revealed consistent local patterns: low A.CRP/C.CRP and lower C3/C4 favored Not-Broken, whereas higher CRP/complement with smaller neck and irregular shape pushed toward Broken classifications. It can be concluded that an interpretable machine learning (ML) pipeline captured clinically plausible, nonlinear interactions between inflammation and aneurysm geometry. Integrating explainable ML with conventional statistics may enhance rupture risk stratification, enable patient-level rationale, and inform personalized management. These results could significantly contribute to the quality of treatment for patients with intracranial aneurysms.

## 1. Introduction

Many people with intracranial aneurysms (IAs), a common cerebrovascular disorder that affects 2–5% of the general population, do not exhibit any symptoms until they rupture [1]. SAH (subarachnoid hemorrhage), a catastrophic event with a mortality rate ranging from 25% to 50%, is caused when unruptured intracranial aneurysms (UIAs) rupture. According to studies, almost half of the survivors experience severe disability after SAH [2]. Due to its prevalence in middle-aged people, SAH contributes significantly to the socioeconomic burden and frequently results in a loss of years of productive life [3]. Many studies have dealt with the development of growth and rupture of UIAs. These studies aim to evaluate the rupture probability and direct decision-making, guaranteeing that high-risk patients receive prompt intervention while avoiding needless procedures for those with low risk [4]. The latest studies are focusing on inflammatory mediators and complement system components that have been found in aneurysm walls and luminal blood, suggesting persistent vascular inflammation [5]. Inflammatory processes weaken the arterial wall of UIAs and increase their vulnerability to rupture by causing endothelial dysfunction, extracellular matrix degradation, and vascular remodeling [6]. Their results demonstrate the importance of inflammation as a therapeutic target in the management of UIAs and research into anti-inflammatory techniques to slow their progression and reduce the risk of rupture [7].

On the other hand, machine learning (ML) has become a potent tool for determining the rupture risk of intracranial aneurysms [8]. While traditional rupture risk assessment techniques depend on predetermined criteria like aneurysm size and location, ML can spot subtle patterns that traditional analysis might miss [9]. Recent research has shown that by utilizing morphologic characteristics like size ratio, aspect ratio, and wall shear stress, ML models such as Random Forest, support vector machines, and neural networks achieve high accuracy in identifying ruptured and unruptured aneurysms [8,10]). Despite their promise, a key challenge remains in ensuring the interpretability of these models for clinical application. Methods such as SHapley Additive exPlanations (SHAP) and Local Interpretable Model-agnostic Explanations (LIME) have been employed to provide insight into feature importance, fostering greater clinician trust in ML-based predictions [11].

Several studies [5,6,7,12,13] examined how inflammatory markers relate to the rupture of intracranial aneurysms. With the use of ML algorithms, we will try to identify connections between inflammatory markers and aneurysms’ morphological characteristics, trying to bridge the gap between the central role of inflammatory mediators and complement system components that have been found in the aneurysm wall and luminal blood, and how these immune signals interact with aneurysm geometry to influence rupture in patient-level data. In order to support clinical decision-making and enhance risk stratification for unruptured aneurysms, we aim to provide interpretable insights into ML predictions. This could facilitate clinical decision-making and enhance risk stratification for patients with UIAs.

This study aims to determine whether an explainable ML model that jointly leverages multi-site inflammatory biomarkers measured in the aneurysm sac, parent artery, and peripheral vein, and detailed aneurysm morphology, can accurately classify rupture status while providing case-level rationales suitable for clinical interpretation. A secondary aim is to explore how inflammatory activity interacts with structural features to illuminate mechanisms that may underlie aneurysm instability. Our contributions are threefold: (i) we demonstrate an ML model that achieves high performance on a small, clinically realistic cohort; (ii) we provide transparent, case-level explanations linking elevated CRP/complement activity with specific geometric patterns (e.g., narrow neck, irregular shape); and (iii) we position an interpretable ML model as a complementary tool to statistical analysis to support clinical decision-making and enhance risk stratification for patients with UIAs.

### Related Work

Machine-learning (ML) approaches have been increasingly applied to aneurysm rupture assessment, generally leveraging morphological and hemodynamic descriptors with model-agnostic explainability. Interpretable pipelines using feature attribution (e.g., SHAP/LIME) show that ML can align with established domain knowledge and highlight geometry-linked risk factors, while outperforming or complementing conventional scores in many cohorts [11,14,15,16]. Several studies on multidimensional data (clinical + morphology ± hemodynamics) report that models such as Random Forests and gradient methods provide competitive discrimination and case-level rationales suitable for clinical discussion [15,17,18]. A large body of work has therefore focused on imaging-derived predictors of rupture, particularly aneurysm morphology.

Inflammation is a recognized contributor to aneurysm pathobiology, and numerous works link circulating or tissue-level markers, particularly C-reactive protein (CRP) and complement components, to aneurysm presence, behavior, or outcomes [13,19,20,21]. Recent translational studies (including proteomic analyses) reinforce inflammatory signatures in intracranial aneurysms, while Mendelian Randomization (MR) analysis connects cytokine/CRP signals to formation and rupture pathways [13,20,21]. Despite this, most ML-based rupture models have not jointly incorporated multi-site inflammatory biomarkers (e.g., systemic, parent-artery, and intraluminal sampling) alongside morphology with explicit per-patient explanations, an integration our study advances.

Against this background, our work contributes to the literature in two ways. First, we integrate multi-site inflammatory biomarker measurements (CRP/C3/C4 and immunoglobulins)—obtained from systemic circulation, parent-artery samples, and intraluminal/aneurysm-sac samples—together with established morphological descriptors, aiming to capture nonlinear relationships and cross-domain interactions between compartment-specific inflammatory biomarkers and aneurysm morphology that are not readily represented by additive linear models or by analyses restricted to a single data modality (e.g., morphology alone or blood biomarkers alone). Second, we emphasize patient-level explainability by combining cohort-level summaries (global feature importance) with case-level explanations (LIME) to support clinically interpretable reasoning about why a specific aneurysm is predicted as ruptured or unruptured, including analysis of misclassified cases in the held-out test set. By positioning explainability as a bridge between model outputs and observable patient feature patterns, our approach is intended as a clinically oriented, interpretable ML framework for rupture risk assessment in a setting where multi-site biomarker acquisition is feasible, but cohort sizes are necessarily limited.

## 2. Materials and Methods

### 2.1. Patient Population

This retrospective study is based on a prospective study conducted between February 2021 and April 2023, in the neurosurgery department, University of Patras, Greece, on patients diagnosed with intracranial aneurysm (IA). With endovascular therapy, the researchers were possible to safely take a blood sample from both the carotid artery and the inside of the aneurysm sac at the same time without interfering with the regular surgical procedure or subjecting the patients to needless surgical procedures. Every patient who signed up for our study is an adult (over the age of 18) with a comparable BMI, co-morbidities, and smoking history. Malnourished patients and those with a history of cancer, autoimmune or inflammatory disorders, liver or kidney disorders, or malnutrition were excluded. Patients receiving immunosuppressive or corticosteroid therapy for a particular illness are similarly affected. The results may be impacted by these drugs and medical conditions due to the immune system disorders they cause. The aneurysms inside the brain were all saccular because this morphology is much more common, and it also made it possible to distinguish between samples taken from the parent artery and those taken from inside the aneurysmal sac [5].

**Figure 1:**
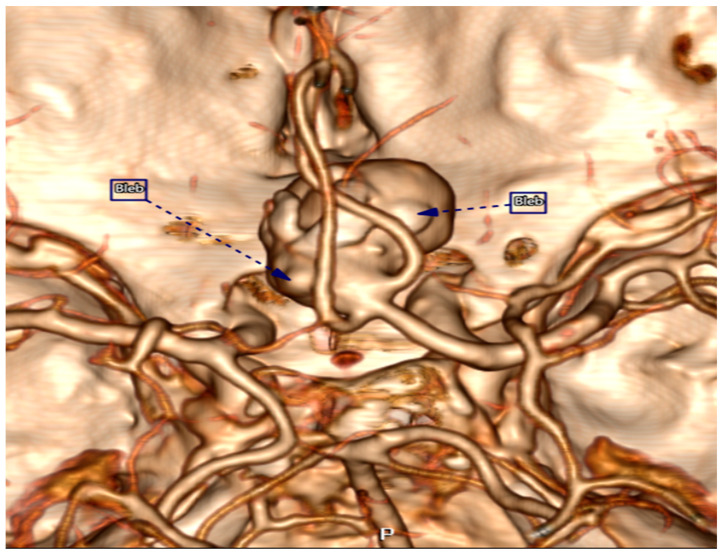
3-D image of an intracranial aneurysm showing the presence of blebs (BLEB).

**Figure 2:**
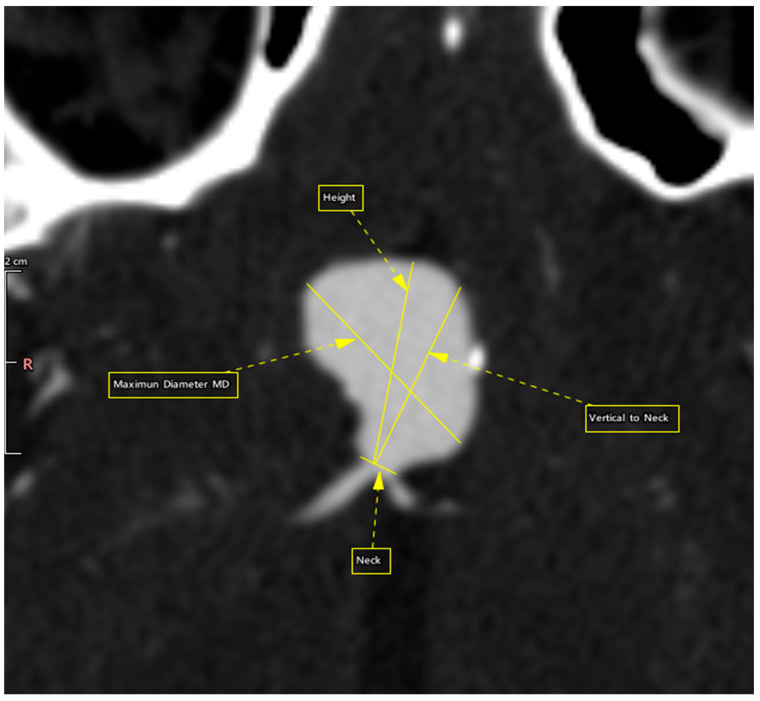
2-D reconstruction of an intracranial aneurysm showing the measurements of numerical features.

All our patients underwent intracranial CTA’s, which were performed on a CANON Aquilion Prime 160 CT scanner (former TOSHIBA) and were processed in the Vitrea Server station before the endovascular therapy and surgical procedure. The protocol began with a non-contrast brain CT, followed by a contrast-enhanced CTA scan triggered by a bolus-tracking technique. A dose of 60–80 mL of iodinated contrast agent was administered at 4 mL/s, tailored to the patient’s cardiac status, using standard acquisition parameters. Out of 36 patients, one was excluded due to insufficient data from brain CTA’s, leaving 35 participants in our retrospective study. Thus, in addition to the original prospective study, our dataset includes morphological characteristics and measurements of the aneurysms obtained from brain CTA’s and had processed in the Vitrea Server station (Figure 1 and Figure 2).

Scientific Review Board and Ethics Committees approved the study, which was carried out in compliance with ethical standards (Re: 37–14/01/2021). The hospital conducted medical research involving human subjects in accordance with the Declaration of Helsinki’s ethical guidelines [5].

### 2.2. Features

The whole dataset comprises clinical and biochemical data from patients with IAs. Key demographic features include sex (SEX) and age (AGE). Anatomical attributes focus on aneurysm location (LOCI), maximum dimension (MD), aspect ratio (AR), neck size (NECK), and vertical distance neck to aneurysm dome (VERTI). Additional morphological characteristics include the presence of blebs (BLEB) and irregular shape (IR.SH). The average age of the patients was 57.1 ± 12.5 years, with women comprising 60% (21 patients) of the group. The mean aneurysm size measured 7.8 ± 4.5 mm. Based on rupture status, the patients were categorized into two groups: 15 had an unruptured intracranial aneurysm (uIA), while 20 had a ruptured intracranial aneurysm (rIA). The additional features are presented in Table 1.

**Figure 3:**
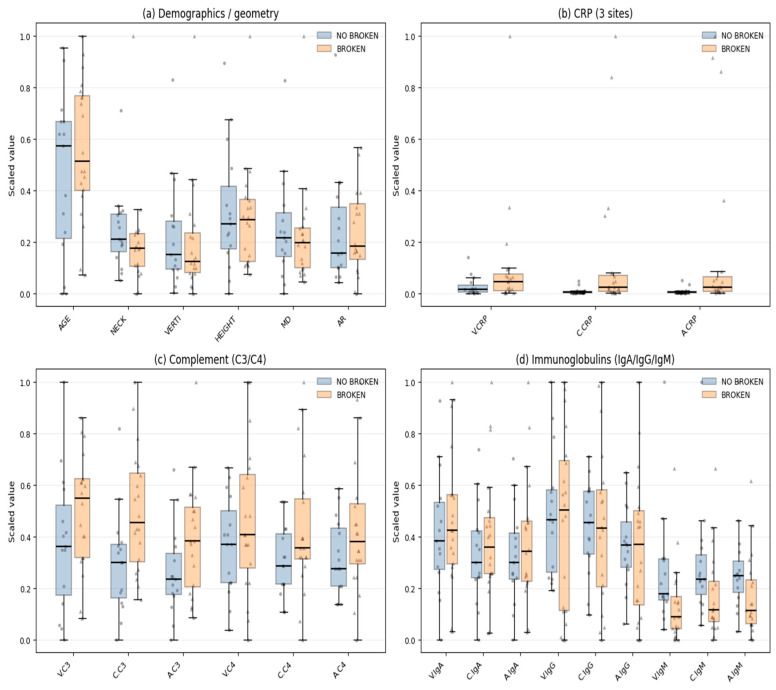
Distribution of numeric features by class Broken: (**a**) Demographics/geometry, (**b**) Vein, Carotid, Aneurysmal CRP, (**c**) Vein, Carotid, Aneurysmal Complement C3/C4, (**d**) Vein, Carotid, Aneurysmal Immunoglobulins (IgA, IgG, IgM).

**Figure 4:**
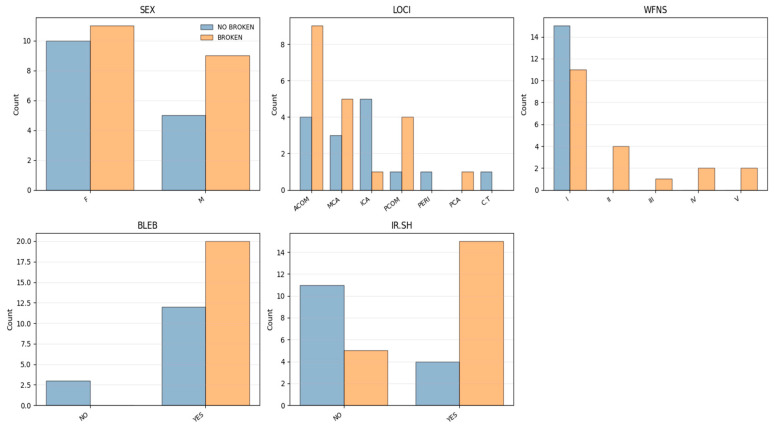
Distribution of categorical features by class Broken (Sex, Aneurysm’s Location/LOCI, WFNS, Aneurysms’ morphology Bleb/Irregular Shape).

The WFNS (World Federation of Neurosurgical Societies) scale is also present, potentially indicating clinical severity. Biochemical data, such as inflammatory and immune-related markers, were measured at different vascular locations, such as the vein, aneurysm sac, and the carotid artery. These markers include CRP (C-reactive protein), complement proteins C3 and C4, and immunoglobulins IgG, IgA, and IgM. Hereafter, they will be referred to in the text as: V.CRP = Vein CRP, C.CRP = Carotid CRP, A.CRP = Aneurysm sac CRP. Likewise, complement proteins C3 and C4, and immunoglobulins IgG, IgA, and IgM. The event of rupture (Broken) serves as a key outcome variable, distinguishing between ruptured and unruptured aneurysms. The dataset features’ distribution is shown in Figure 3 and Figure 4.

### 2.3. Features Engineering

Each feature used in a machine learning algorithm must contribute equally to the final prediction. However, the features differ in range and unit, hence affecting their importance into a ML algorithm. Feature engineering or feature scaling is a preprocessing step that involves transforming numerical values of the features to a common scale. That preprocessing is aiming to normalize the range, distribution, and magnitude of features, reducing potential biases and inconsistencies, ensuring accurate and efficient model training and performance. Common techniques include standardization and normalization [22]. Min–max normalization for numerical features was chosen for this study to scale the values of a feature to a range between 0 and 1.

Additional preprocessing techniques are needed to convert categorical variables because most machine learning models only accept numerical variables [23]. One-hot encoding, a method for converting categorical variables into a binary format, was chosen for this study. It creates new binary columns (0 and 1) for each category in the original variable. Each category in the original column is represented as a separate column, where a value of 1 indicates the presence of that category, and 0 indicates its absence. Using the command drop_first = True, every column of categorical variables is represented as follows: BROKEN_YES (TRUE, FALSE), IR.SH_YES (TRUE, FALSE) etc.

**Figure 5:**
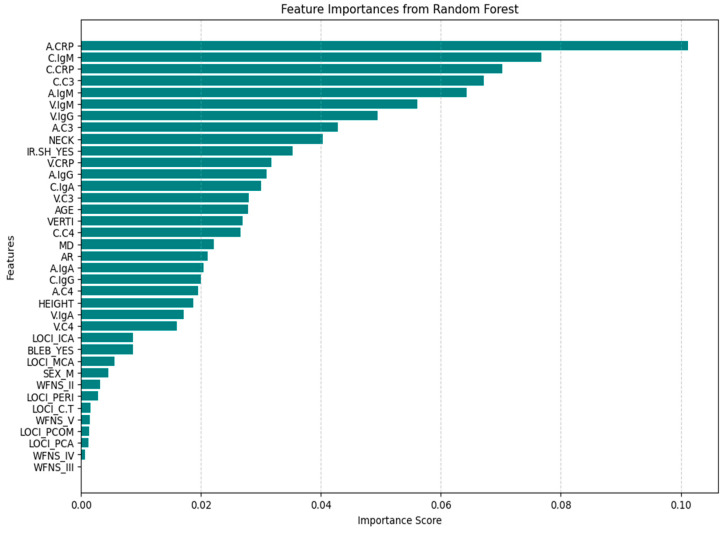
Tree-based feature selection with RF feature importance tells us which features are more important in making an impact on the target feature.

Removing redundant and unnecessary features from a dataset is crucial for ML algorithm performance. With the help of feature selection techniques, we can choose pertinent features, lower dimensionality, use less data for learning, increase predictive accuracy, and improve the built model’s overall performance. Feature selection plays a significant role in identifying irrelevant and redundant features from a dataset [24]. There are several types of feature selection techniques, such as filter methods, wrapper methods, embedded methods, etc. For this study and from Embedded methods, Tree-based feature selection with Random Forest was executed to whole dataset before testing and choosing the best model. This method helped us to find which features are more important (Figure 5).

After examining feature importances (Figure 5) and distribution features by class (Figure 1), we removed the features with little or no importance, such as ‘AGE’, ‘WFNS’, ‘LOCI’, and ‘BLEB’. Although it is middle-ranked, the AGE feature distribution by rupture status (Figure 1) shows significant overlap between classes and adds redundancy rather than new information. The WFNS (World Federation of Neurological Surgeons Scale) is used clinically to assess patients after rupture has occurred, meaning it does not help predict rupture but rather describes its impact. If WFNS is included, we could introduce data leakage. The feature importance plot shows different locations (LOCI) individually ranked low, meaning the location does not contribute much to the prediction. The BLEB feature has a very low importance score, suggesting that its presence does not strongly differentiate ruptured from unruptured aneurysms. Feature retention/removal was guided by (i) embedded RF feature importance, (ii) inspection of class-wise distributions, and (iii) clinical plausibility and leakage risk. Variables with negligible contribution and/or post-rupture clinical meaning (e.g., WFNS) were removed to reduce redundancy and prevent leakage. By contrast, we retained V.C4 and V.IgA because they are biologically meaningful components of the systemic complement and humoral immune response panels and were not considered redundant in the multi-site inflammatory profiling framework used in this study.

### 2.4. ML Algorithms

The methodology for this research involved the implementation of five different machine learning algorithms to create five corresponding models. The selected algorithms were Decision Tree, Adaptive Boosting (AdaBoost), Gradient Boosting Machine (GBM), Extreme Gradient Boosting (XGBoost), and Random Forest. This selection is justified by their extensive use within the research community and their documented success in various medical domains, such as automated medical diagnosis, brain cancer analysis, and the diagnosis of conditions like acute bronchiolitis, breast cancer, diabetes, hypertension, Primary Hyperparathyroidism, and Alzheimer’s disease [25,26,27,28,29,30].

One non-parametric supervised learning algorithm that is used for both regression and classification problems is the decision tree (DT). Branches, internal nodes, leaf nodes, and a root node make up its hierarchical tree structure. It is a popular tool for medical researchers who want to find and take advantage of patterns and relationships among a lot of variables, and predict the cause of a disease [26,31].

In 1997, Freund and Schapire presented the AdaBoost (Adaptive Boosting) machine learning algorithm, which is based on the idea of boosting, a well-known ensemble learning technique that attempts to increase classification accuracy by combining several weak classifiers into a strong classifier. Using the same training set, the algorithm iteratively trains various weak learning algorithms before combining these weak learners to create a more potent final classifier. An ensemble classifier that is stronger and more efficient than any single classifier is produced by successively training weak classifiers. The diagnosis of diabetes, hypertension, Alzheimer’s disease, and several types of cancer are examples of its clinical applications [27,32].

GBM (Gradient Boosting Machine) is a method that builds a predictive model as a stage-wise additive ensemble of weak learners, most often small decision trees, by minimizing a chosen loss function via gradient descent in function space. Jerome H. Friedman introduced Gradient Boosting Machines in his 2001 paper, “Greedy Function Approximation: A Gradient Boosting Machine [33].”

Praised for its speed and scalability with large datasets, XGBoost (Extreme Gradient Boosting) is a powerful supervised learning algorithm. Its core mechanism follows the gradient boosting principle: it builds a strong predictive model by sequentially adding weak decision trees, each one trained to correct the mistakes of the current ensemble. This optimized implementation, originally created by Tianqi Chen from the University of Washington, delivers this advanced functionality with remarkable computational efficiency [34].

Random Forest (RF) is an ensemble algorithm that aggregates the predictions of multiple decision trees to produce an outcome and to enhance predictive accuracy. Both regression and classification issues can be handled by it. The output of regression tasks is the average of all the trees’ predictions, while in classification tasks, the final prediction is decided by a majority vote among the trees that it generates. Even though individual trees can make mistakes, their collective choices reduce errors and produce predictions that are more solid and trustworthy [35].

### 2.5. Oversampling and Optuna Study

Dataset imbalance is a common problem in classification tasks. Artificially increasing the instances of a minority class helps to offset its underrepresentation. This technique is called oversampling and is essential for the creation of models where the minority class is crucial, like in medical diagnostics. Random oversampling [36], SMOTE [37] and ADASYN [38] are three well-known techniques that were used and assessed using the dataset. Random oversampling was tested and was found to be the most effective technique for optimizing model performance in this specific dataset.

One of the easiest and most popular oversampling techniques is random oversampling. To balance the classes, it entails randomly reproducing instances of the minority class until it hits a predetermined threshold. Despite its drawbacks and propensity for overfitting, random oversampling is an easy and efficient method for dealing with unbalanced datasets [36]. Random oversampling, for instance, has been used to boost the precision of credit card fraud detection models, forecast early Alzheimer’s disease symptoms, and forecast subject depression [25].

**Figure 6:**
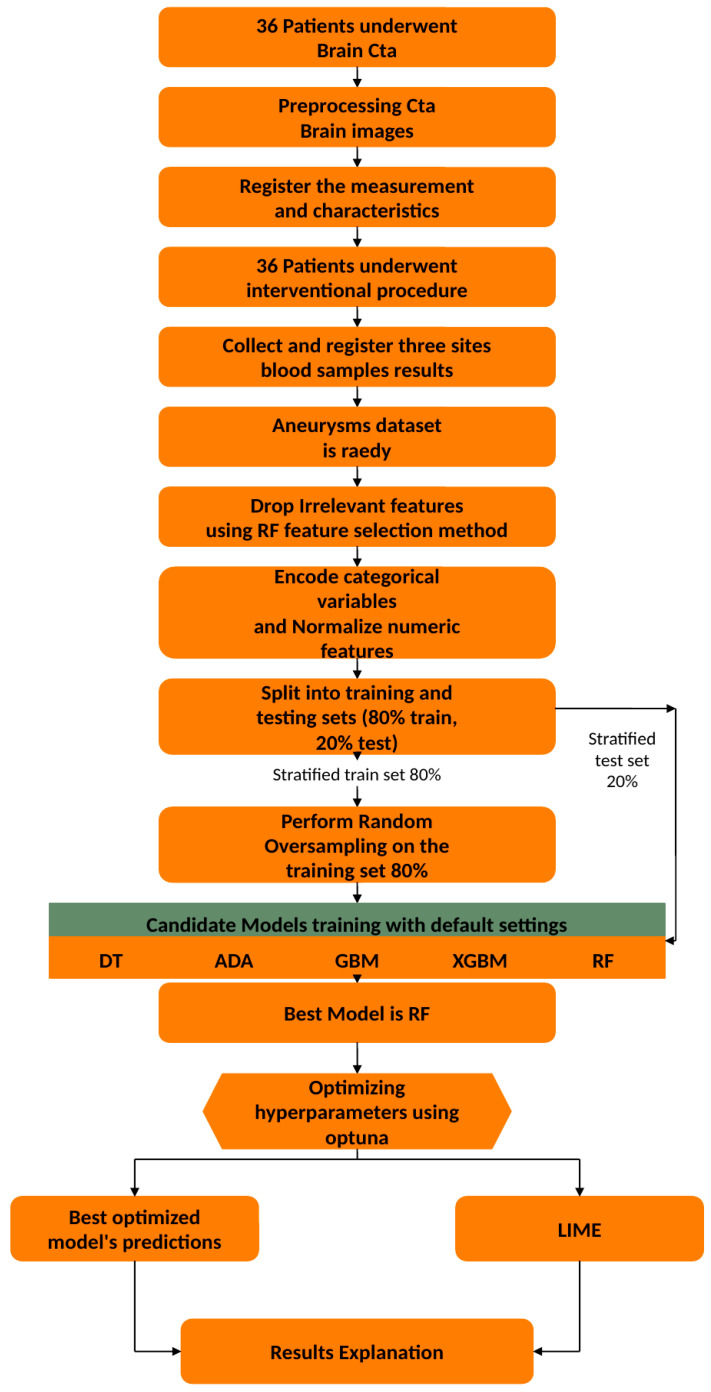
The steps of this study.

Optuna study is a novel and open-source tool for automatic hyperparameter search and optimization that can be used with any machine learning algorithm. Its core strength is a collection of potent features: it uses cutting-edge sampling and pruning algorithms to effectively search the space, and its architecture is scalable, allowing for parallelization and integration with cloud backends such as Kubernetes. Moreover, Optuna improves usability with a small code footprint and add-on tools like the Optuna Hub, which provides access to a library of community-contributed features, and the Optuna Dashboard, which analyzes experiments [39]. In Figure 6, the study’s comprehensive methodology and all of its phases are displayed.

### 2.6. Evaluation Metrics

Accuracy has been regarded as the most significant metric in many studies, and is the proportion of correct predictions (TP+TN)/(TP+TN+FP+FN). Here, TP denotes true positives, TN true negatives, FP false positives, and FN false negatives. While intuitive, it can be misleading with class imbalance: a classifier that always predicts the majority class may score high accuracy yet fail to detect the minority class. Accuracy is also threshold-bound and does not reflect how well scores rank positives above negatives [40]. In our dataset, one class is more common than the other; the prediction model could be trained to accurately predict the prevalent class while making poor predictions for the unusual. So, the model’s overall accuracy does not fairly represent this flaw.

On the other hand, the receiver operating characteristic (ROC) curve, sensitivity, specificity, and confusion matrix are typically the most crucial metrics when working with imbalanced datasets. These metrics could lead to a lower overall accuracy when using oversampling techniques. However, they do produce a more broadly trained estimator that is ultimately better suited to classify all instances rather than just the most common class [25].

ROC–AUC. The ROC curve plots true-positive rate (TPR) versus false-positive rate (FPR) across all thresholds. AUC equals the probability that a randomly chosen positive receives a higher score than a randomly chosen negative, thus evaluating ranking performance independently of any threshold and of monotonic score transforms [41]. ROC–AUC is relatively insensitive to prevalence shifts, which aids fair comparison across experiments. However, with extreme imbalance, it can look optimistic, so it is best paired with class-aware metrics or PR analysis [42,43,44,45]. For our model evaluation, ROC–AUC is attractive because (a) it is threshold-independent, summarizing separability over all cutoffs; (b) it is relatively robust to prevalence, enabling comparisons across folds; and (c) it has a clear probabilistic interpretation as pairwise ranking of positives above negatives [40,41,42,43,44,45]. Thus, we report ROC–AUC as the primary selection metric and accompany it with sensitivity/specificity at clinically/operationally relevant thresholds, plus PR curves for completeness [42,43,44].

Much like the ROC curve, another curve is used for the evaluation of classification algorithms. The precision–recall curve shows how precision and recall are balanced at different thresholds, rather than a single value (e.g., accuracy, f − 1 score, etc.). Higher recall and precision levels are indicated by a larger area beneath the curve. While high recall is attained by limiting the occurrence of false negatives within the pertinent outcomes, high precision is found by minimizing the number of false positives among the results. High scores in both metrics show that the classifier is successfully producing accurate results [42,44,45].

Sensitivity and Specificity. Sensitivity (recall, TPR) is TP/(TP+FN), capturing how many true positives are found. Specificity (precision, TNR) is TN/(TN+FP), capturing how many true negatives are correctly rejected. A model’s performance can be assessed using two crucial metrics: specificity and sensitivity. This is particularly true for classification scenarios that involve two classes altogether and can reveal how well the model distinguishes between the first and the second class [25,40,45].

Confusion matrix. The confusion matrix tabulates TP, TN, FP, FN, and underlies most discrete metrics: accuracy, precision/recall, and specificity. It both summarizes and visualizes how well a model distinguishes classes. When dealing with binary classification problems like the one in this work, a special case of a 2 by 2 confusion matrix is frequently used and it is formed by the TP, TN, FP, and FN fields [25]. A confusion matrix of this kind looks like this in Table 2:

Another technique called OOF (out-of-fold) validation is used to ensure the evaluation and performance of our model. This method entails splitting a dataset into folds or subsets. After that, one validation set is created for each fold, and the remaining folds are used for training. By repeating this procedure, metrics such as accuracy or error rates are used to evaluate the model’s performance. The sum of the outcomes from every validation run offers a thorough assessment of the model’s data generalization capabilities. This validation technique plays a role in the detection of overfitting, for optimization model hyperparameters, for the identification of base models for ensemble learning, and finally ensures the reliability and accuracy of machine learning models in real-world scenarios [46].

### 2.7. Results Explanation

The explanation of the machine learning algorithm is the next step of our study. An interpretable machine learning algorithm is one where the relationship between the input features and the model’s predictions is clear and understandable to humans [47]. Explainable AI seeks to unravel the “black box” nature of many predictive models. It is crucial, especially in medicine, where trust and compliance require explainability. Without it, ML systems may be seen as unreliable and fail to meet regulatory standards [25].

Interpretability can be divided into ‘Global’ and ‘Local’. Global interpretation is about understanding the model’s decisions, as it gives us a holistic view of its features. That helps to understand the distribution of the target outcome based on the input features [48]. A second key requirement is Local fidelity. Explanations must faithfully reflect how the model behaves near the instance being explained, even if they are not globally faithful; global importance can diverge from local importance, and producing globally faithful yet interpretable summaries for complex models remains difficult. Because many state-of-the-art classifiers are opaque, explainers should be model-agnostic, treating the predictor as a black box to ensure broad applicability now and in the future [49].

In order to explain the selected black-box machine learning prediction model, this study takes matter two primary analytical techniques: Random Forest feature importance and LIME. The feature importance in Random Forest (Global interpretation) can be determined using a metric called Gini importance. It measures the total reduction in the Gini impurity of the dataset when a particular feature is used for splitting. The higher the Gini importance, the more important the feature is for the model [50].

LIME (Local Interpretable Model-agnostic Explanations) is a novel technique that explains the prediction of any classifier, and instead of providing a global understanding of the model on the entire dataset, LIME focuses on explaining the model’s prediction for individual instances [49].

A diagnostic and exploratory tool that helps to summarize data and for advanced analyses is the correlation matrix. A correlation matrix is a table that displays the correlation coefficients for a set of variables. The correlation matrix is a table where each entry represents a correlation coefficient, with values of 1 indicating a strong positive association between variables, 0 indicating no association, and −1 indicating a strong negative association. There are different methods for correlation analysis: Pearson correlation, Kendall rank correlation, and Spearman correlation analysis. Spearman is the suitable method for our dataset because Spearman correlation handles nonlinear relationships, works with different data types, is outlier-resistant, and requires no distributional assumptions [51,52].

## 3. Results

This project was carried out in a Windows 11 environment, specifically a 11th Gen Intel Core i5 CPU and 8GB RAM. For data processing with machine learning algorithms, we used ANACONDA’s DISTRIBUTION PLATFORM Jupyter Notebook (version 7.2.2), and all tools and libraries obtained from scikit-learn Machine Learning in Python (version 3.11.9). All open sources. In an automated and powerful environment, the capabilities of these libraries were able to produce robust and reliable results.

### 3.1. Comparison of the Five Models

**Figure 7:**
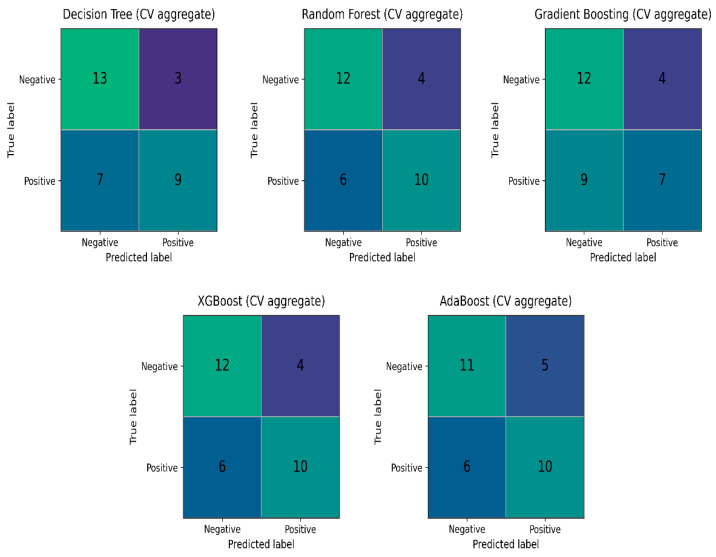
Aggregated confusion matrices for the five evaluated ML models. The results reflect the cumulative performance across all stratified five-fold cross-validation iterations.

Due to the small number of cases, the stratified cross-validation (Skf-CV) technique was chosen to evaluate the performance of five algorithms on unseen data during testing. This technique is used in machine learning to make sure that the class distribution across the entire dataset is maintained across each fold of the cross-validation process. When working with small, unbalanced datasets where some classes might be underrepresented, this is especially crucial [53]. In this study, performance evaluation on the training set was utilized primarily as a diagnostic framework during the model development phase. Monitoring training metrics allowed us to assess the models’ learning capacity and identify potential issues such as underfitting, data leakage, or misaligned labels [54]. While real-world performance is estimated through the held-out test set and cross-validation, these internal diagnostics were crucial for ensuring the stability of the algorithms, given the technical complexity of the multi-site intravascular features. The above-mentioned five algorithms were tested with their default settings, with random oversampling (ROS) on the training set and five-fold Skf-CV, with the results seen in Table 3 and their aggregate confusion matrices in Figure 7.

The totals in the aggregate confusion matrices sum to 32 rather than 35 because these matrices were computed on the resampled training data used during cross-validation, not on the full cohort. After the train/test split, the training set contained 28 cases (16 Broken and 12 Not-Broken). Random oversampling was applied on the training test to mitigate class imbalance by duplicating minority-class observations, bringing the effective training size to 32 with a balanced distribution (16 Broken and 16 Not-Broken). Consequently, the aggregated confusion matrices reflect 32 training instances (including duplicated samples), whereas the remaining cases from the original 35-case dataset belonged to the held-out test set and were evaluated separately without resampling.

As shown in Table 3 and the aggregate confusion matrices (Figure 7), Random Forest (RF) emerged as the most promising algorithm. The receiver operating characteristic (ROC) curve summarizes a classifier’s performance across decision thresholds by plotting the true-positive rate against the false-positive rate, while the area under the ROC curve (ROC–AUC) quantifies the model’s ability to discriminate between the two classes (ruptured vs. unruptured) [43]. RF achieved a mean ROC–AUC of 0.903 (SD 0.095), indicating strong discrimination and comparatively stable performance across cross-validation folds. Although RF and XGBoost show identical aggregate confusion matrices in Figure 7, Table 3 indicates that RF attains a higher ROC–AUC with lower variability than XGBoost, supporting its selection as the classifier. In addition, RF is an ensemble method that combines predictions from multiple decision trees via majority voting, which generally improves robustness compared with a single classifier.

### 3.2. RF’s Results

In this section, we will describe the steps for our model’s improvement and the results. Starting from scratch, Random Forest (RF) was trained and tested with its default settings and without oversampling. The results are seen in Table 4.

The next step for the improvement of our model, some additional techniques, such as Optuna study and Oversampling. Random oversampling balanced our dataset as the minority class was 15 unruptured to 20 ruptured aneurysms. The results of RF after random oversampling and Optuna tuning are more balanced and are seen in Table 5.

For further validation of the performance of our RF model, a technique called OOF (out-of-fold) validation is used. The out-of-fold accuracy of our model is 0.8438. An OOF accuracy of 84.38% suggests that our model performs well during cross-validation and generalizes well to unseen data. If there is a significant drop in accuracy when tested on the test set, it could indicate overfitting or issues with the data. Such a problem does not exist because the final test accuracy is 86% (Table 5).

**Figure 8:**
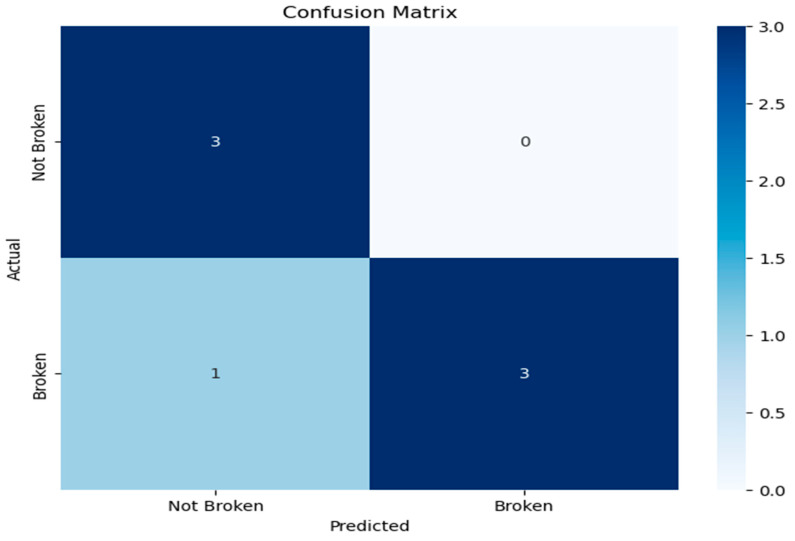
In the confusion matrix, one ruptured aneurysm of our test set was misclassified as unruptured (false-negative).

Other metrics that were used for the RF model’s evaluation, such as the confusion matrix, area under the ROC curve (AUC–ROC), and precision–recall (PR) curve, gave a clear image of our model’s performance. Precision and recall metrics can be calculated from the confusion matrix. The confusion matrix is seen in Figure 8.

**Figure 9:**
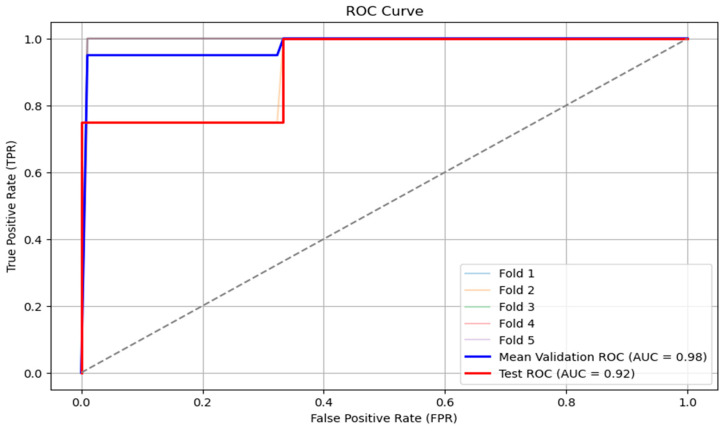
The results for the ROC-AUC score for each fold used for cross-validation.

The receiver operator characteristic (ROC) is a probability curve that plots and visualizes model performance at various threshold values. The area under the curve (AUC) is the measurement of the ability of a classifier to distinguish between classes. RF performs well, achieving an excellent ROC-AUC (97%). The results for ROC-AUC score were train ROC AUC 0.97, validation ROC AUC 0.98, and test ROC AUC 0.92. An outcome 97% of train ROC AUC suggests the model has a strong ability to distinguish between positive and negative classes. Validation performance (98%) that is almost identical to the training performance suggests that the model generalizes well and does not overfit to the training data. The test ROC-AUC is also high at 92%, indicating that the model maintains a strong ability to distinguish between classes on unseen data (Figure 9).

**Figure 10:**
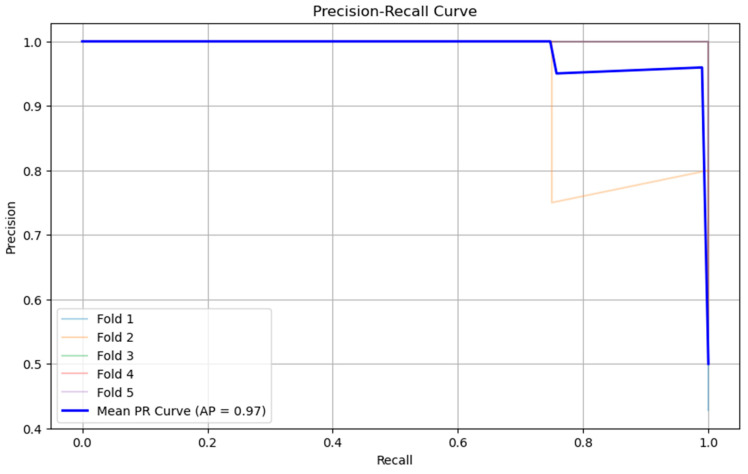
Precision–recall curve with AP = 0.97.

The precision–recall (PR) curve evaluates a classifier across all decision thresholds, showing the trade-off between recall (how many true positives are found) and precision (how many predicted positives are actually correct). A larger area under this curve means the model maintains both high recall and high precision over many thresholds. High recall comes from avoiding false negatives, and high precision comes from limiting false positives. In Figure 10, we present RF’s precision–recall curve.

In imbalanced problems, PR and especially average precision (AP) are more informative than accuracy. Here, the AP of 0.97 indicates that the model separates ruptured from unruptured aneurysms extremely well across thresholds.

The classification report is presented in Table 6.

On the test set, sensitivity (TPR) and weighted average sensitivity are 0.75 and 0.86, respectively. Specificity (TNR) and weighted average specificity are 1.00 and 0.89, respectively. This means the model correctly identified 75% of truly ruptured cases (3/4) but missed 25% (1 false negative), while it perfectly ruled out all non-ruptured cases (0 false positives; 3/3 correct).

### 3.3. Global Interpretation of RF’s Results

**Figure 11:**
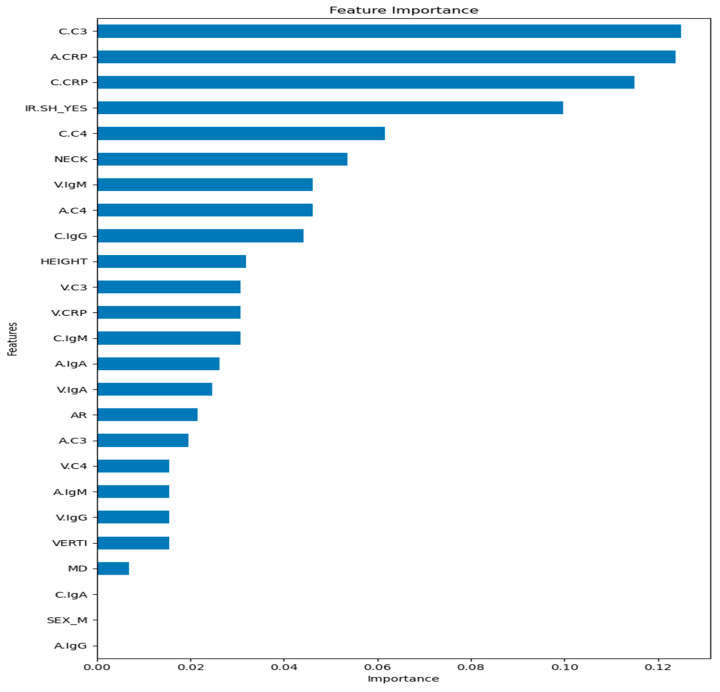
Feature importance of RF (final model).

The feature importance of RF (optimized model) represents how much each feature contributes to the model’s predictions. Higher importance values indicate a stronger influence on decision-making (Figure 11).

The most important features in our model for the correct discrimination between classes are C.C3 (carotid C3), A.CRP (aneurysmal CRP), C.CRP (parent artery CRP), and IR.SH (shape of the aneurysm), C.C4 (parent artery C4), and NECK (aneurysm’s neck width). All these features are likely to have the strongest correlation or relationship with the target variable (Broken/Not-Broken).

The middle-ranking features include V.IgM, A.C4, C.IgG (various immunoglobulins and complement factors), height (height of the aneurysms), V.C3, V.CRP (vein blood C3 and CRP), C.IgM (carotid blood immunoglobulin M), A.IgA, and V.IgA (aneurysmal and vein blood immunoglobulin A, antibody response). These features are likely to have a moderate correlation or relationship with the target variable.

The lower-importance features, such as AR, A.C3, V.C4, A.IgM, VERTI, MD, SEX (male, female), and A.IgG, C.IgA (aneurysmal and vein immunoglobulin measurements), have minimal influence on prediction.

Summarizing, the results suggest that a combination of immune response markers (C3, CRP, C4) and aneurysm morphology (neck, irregular shape) is the primary driver of aneurysm risk of rupture in our dataset.

### 3.4. Local Interpretation of RF’s Results

A tool that explains the model’s decision boundary in a human-understandable way. The name of this magical library is LIME. LIME (Local Interpretable Model-agnostic Explanations) is a novel explanation technique that explains the prediction of any classifier in an interpretable and faithful manner by learning an interpretable model locally around the prediction. Instead of providing a global understanding of the model on the entire dataset, LIME focuses on explaining the model’s prediction for individual instances.

Table 7 presents the final classification (true/false for ‘broken’) from the Random Forest (RF) model, along with the probability score that informed that decision. A probability threshold of 0.5 was applied, meaning instances with a probability of 0.5 or higher are classified as “Broken” (True), while those below are classified as “No” (False).

**Figure 12:**
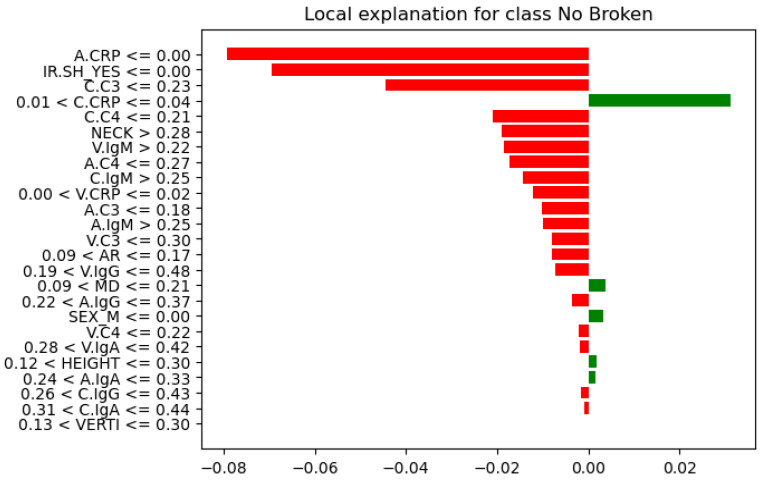
Instance index 31 (local explanation of correctly classified as Not-Broken class and its features).

**Figure 13:**
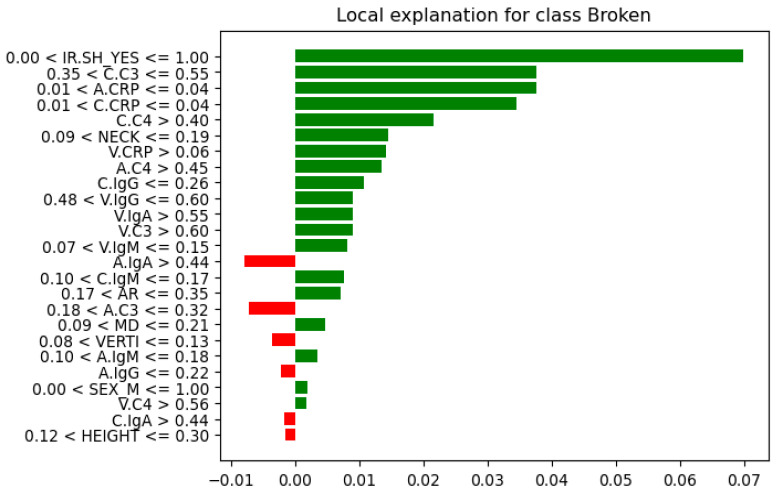
Instance index 3 (local explanation of correctly classified as the Broken class and its features).

**Figure 14:**
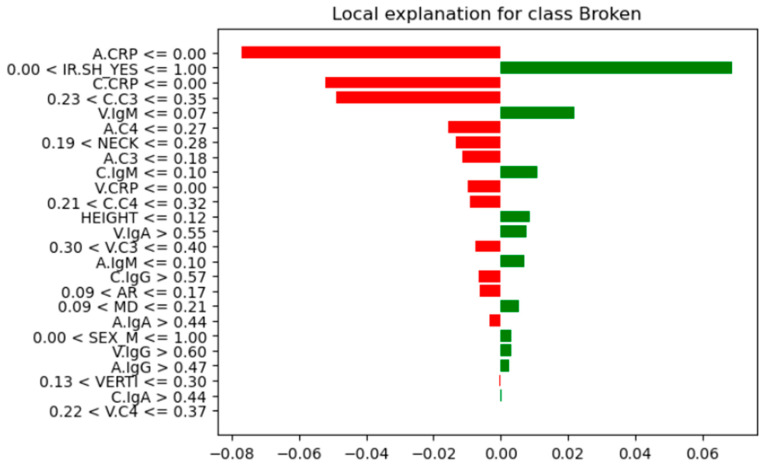
Instance index 11 (local explanation of the misclassified instance as the Not-Broken class and its features).

Further analysis of feature instances that contributed most to their correct classification was needed to identify differences, similarities, and patterns between the Not-Broken and Broken cases. Here comes the LIME explainer. From these seven instances (Table 7), we present the one indexed 31 with the highest probability of being classified as Not-Broken, the one indexed three with the highest probability of being classified Broken based on their features as they were produced from LIME, and the wrong classification indexed 11 (Figure 12, Figure 13, and Figure 14, respectively).

For these seven test-set instances, LIME explainer identified the following: across the cases that share common measurements, arterial and carotid CRP (A.CRP, C.CRP) at or near zero consistently aligned with the Not-Broken class, whereas non-zero CRP values pushed toward Broken. C3 and C4 showed a similar pattern: lower levels supported Not-Broken, while higher levels and most prominently venous C3/C4 and carotid C3 were frequently associated with Broken. Immunoglobulins were less consistent overall; venous IgA/IgG tended to be higher in Broken, while arterial IgA/IgM could be lower in Broken or moderate, making their direction weaker to moderate compared with CRP and complement. AR occasionally appeared high in Not-Broken and was not decisive. HEIGHT tended to be higher in Not-Broken, but its effect was modest. Smaller NECK and smaller VERTI values were characteristic of Broken. Consistent with that, higher V.C3 and V.C4 also pushed toward Broken. SEX(M/F) appeared in both classes with no clear predictive pattern. IR.SH (irregular shape = True) was frequently accompanied by Broken, but it was supportive rather than sufficient on its own. A synopsis of these class differences appears in Table 8.

**Figure 15:**
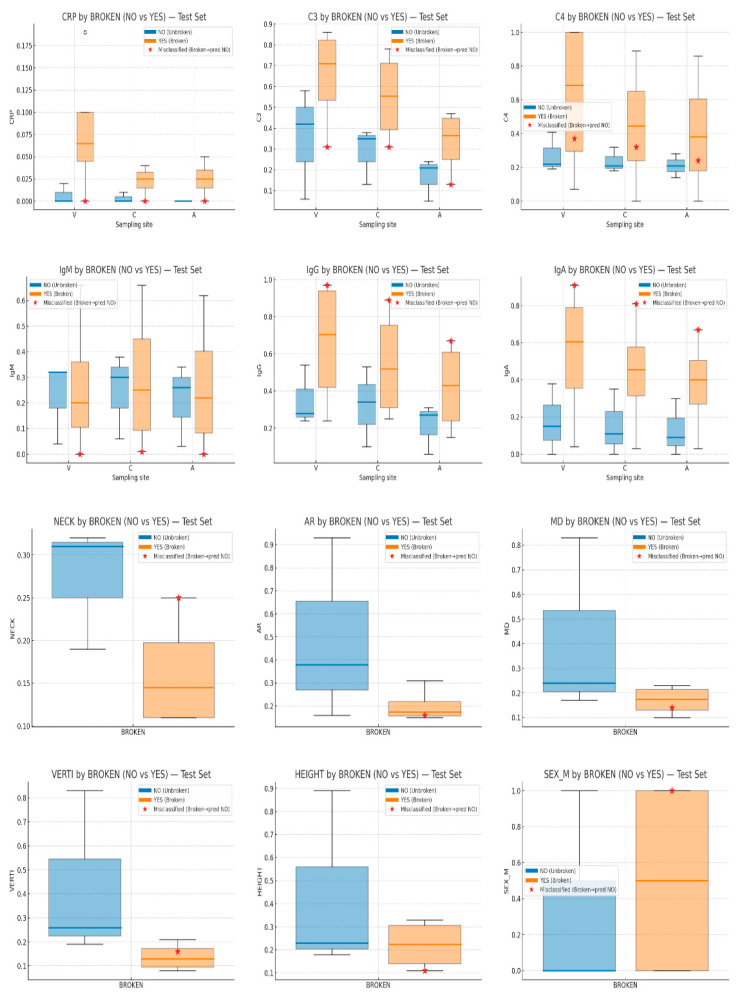

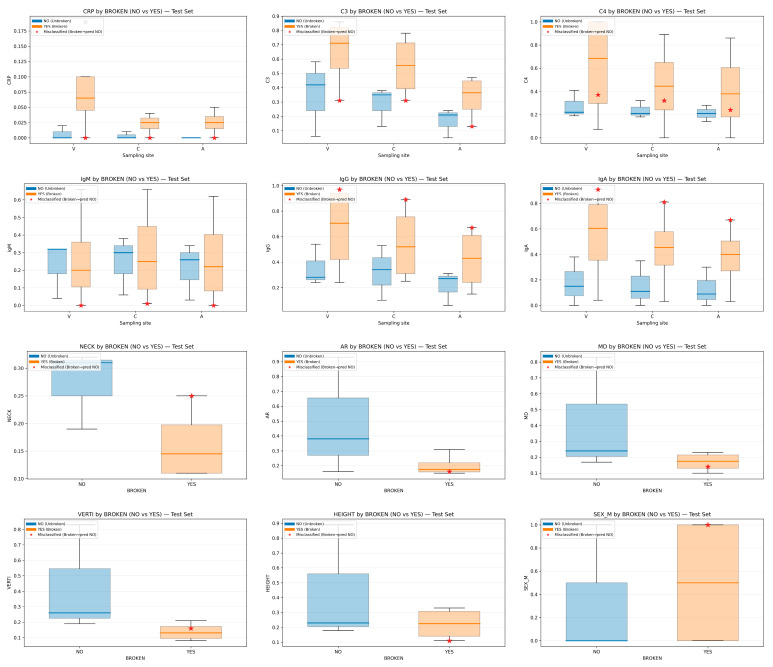
Descriptive distributions of input features in the held-out test set used for case-level explanation (LIME) (V = vein, C = carotid, A = aneurysmal sac).

To contextualize the case-level LIME, we provide a descriptive visualization of the feature distributions for the held-out test instances (Figure 15), highlighting misclassified cases to facilitate interpretation; no statistical inference is drawn due to the small test-set size. After plotting their features in relation to their rupture status (box plots, Figure 15), CRP levels (A.CRP, C.CRP, V.CRP) clearly increased in Broken and remained low in Not-Broken, indicating a strong role in classification. Many theories have been postulated to explain why inflammatory markers such as CRP play a crucial role in aneurysm formation, growth, and rupture [6,7]. Likewise, there was a distinct separation in C3/C4 across sites: lower in Not-Broken and higher in Broken, consistent with immune activation. IgM showed a wider distribution and was not uniformly elevated, whereas IgA and IgG were often higher in Broken. The role of complement activation in aneurysm pathology has been the subject of numerous studies, which provide evidence that immune mechanisms play a major role in rupture risk [5,6]. Wider necks and larger aneurysm dimensions (VERTI, MD, HEIGHT) were more prevalent in Not-Broken, which is consistent with a lower rupture risk according to geometric features that matched the plots [52,53,54]. SEX did not exhibit a distinct classification pattern. Regarding the misclassified instance, it had low CRP, placing it closer to the Not-Broken profile and contributing to the error. C3/C4 were low-to-moderate, further blurring the boundary, and IgA/IgG were extremely high, suggesting the model underweighted these signals relative to CRP/complement.

**Figure 16:**
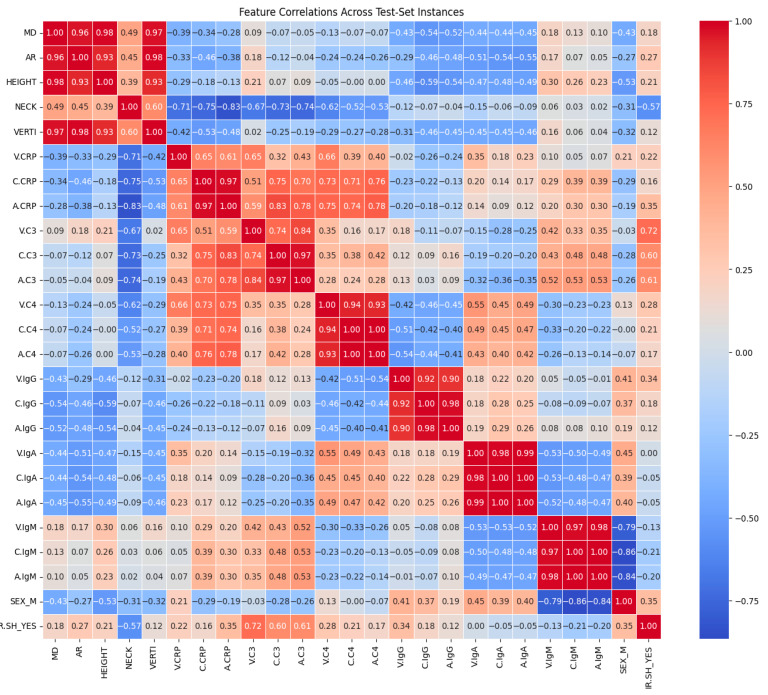
Feature correlations across test-set instances.

Correlation matrix (Figure 16), a descriptive statistical tool that describes the strength and direction of the linear relationship between multiple pairs of variables, confirms both RF feature importance and LIME explainer for the inflammatory core made of parent-artery C3 (C.C3) and CRP in the aneurysm sac and parent artery (A.CRP, C.CRP). In the RF feature importance plot (Figure 8), these three markers appear among the strongest overall predictors of rupture. The correlation matrix shows that BROKEN cases are clearly positively related to V.C3, C.C3, and A.C3, as well as to CRP at all three sampling sites (correlation coefficients of about 0.58–0.72), indicating that higher complement and CRP levels are associated with ruptured aneurysms in this small test set. Consistently, the LIME explainer, which describes the model’s behavior for each of the seven test cases, frequently highlights rules such as C.C3 > 0.35–0.55 and C.CRP/A.CRP > 0.04 as conditions that push the prediction toward the “Broken” class, whereas values below these ranges support a “Not-Broken” prediction.

Also, the correlation matrix confirms RF feature importance and LIME explainer results for the morphological instability captured by irregular shape (IR.SH) and the neck size (NECK). In the RF feature importance plot (Figure 11), IR.SH is the most important non-biochemical feature, and NECK is the next most important geometric feature, just after C.C4. The correlation matrix shows that IR.SH has a moderate positive correlation with BROKEN_YES (~0.47), meaning that aneurysms labeled as irregular are more likely to be broken. NECK, on the other hand, has a strong negative correlation with BROKEN_YES (≈−0.72), indicating that aneurysms with a smaller neck are more often broken in these seven test cases. The LIME plots tell the same story: when IR.SH_YES = 1 it appears high among the influential features and supports the “Broken” prediction, while small neck values (for example, 0.09 < NECK ≤ 0.19 or NECK ≤ 0.28) push the model towards “Broken”, and larger necks push it towards “Not-Broken”.

Furthermore, the correlation matrix confirms RF feature importance and LIME explainer results for the role of IgM and the additional contribution of C4 and IgG. In the RF feature importance plot (Figure 11), venous IgM (V.IgM) and, to a lesser degree, carotid IgM (C.IgM), are among the most important features, along with C4 in the parent artery and aneurysm (C.C4, A.C4) and carotid IgG (C.IgG). The correlation matrix shows that BROKEN_YES is strongly negatively correlated with V.IgM and C.IgM (around −0.7), but positively correlated with C4 and C.IgG. This means that ruptured aneurysms in this test set tend to have lower IgM levels in the blood, but higher levels of complement C4 and IgG. LIME support the same pattern: in cases predicted as Broken, very low IgM values (for example V.IgM ≤ 0.07–0.10 or C.IgM ≤ 0.10–0.17) are among the factors pushing the prediction toward “Broken”, whereas higher IgM values pull several cases towards “Not-Broken”; at the same time, higher C4 and C.IgG values contribute in favor of the “Broken” class.

## 4. Discussion

In a single-center cohort of 35 saccular intracranial aneurysms (IAs) in 35 patients (≈57% ruptured), we developed an interpretable Random Forest (RF) classifier. RF classifier integrates multi-site inflammatory biomarkers (CRP, C3, C4, immunoglobulins) with additional detailed morphometrics (e.g., NECK width, irregular shape, aspect ratio). Our target was to assess the risk of rupture status, including these additional features, and analyze their importance using Global and per-instance (Local) explanations from an RF classifier. The addition of these extra features appears to extend beyond the findings of the statistical studies [5,54,55], providing novel insights into the relationship between aneurysm structure and biochemical markers. Model development addressed class imbalance with random oversampling and optimized hyperparameters with Optuna. Across stratified cross-validation (CV), the tuned RF achieved high discrimination (CV ROC–AUC ≈ 0.98 with accuracy ≈ 0.85). Generalization to the held-out test set remained strong (test ROC–AUC ≈ 0.92; accuracy ≈ 0.86; AP = 0.97). Out-of-fold (OOF) accuracy was ~0.84. The test confusion matrix showed one false negative (sensitivity 0.75; specificity 1.00).

Global feature importance, LIME explainer results on the seven test-set instances, and the correlation matrix converged on a consistent ranking of predictors. The dominant contributors were inflammatory and complement markers—particularly parent-artery C3 (C.C3) and CRP in the aneurysm sac and parent artery (A.CRP, C.CRP), followed by C4—alongside morphological instability (irregular shape, IR.SH_YES) and neck diameter (NECK). BROKEN status showed strong positive correlations with V/C/A-CRP and V/C/A-C3, and negative correlations with NECK, while LIME repeatedly identified high C.C3, elevated CRP, irregular shape, and small neck width as key conditions pushing predictions toward the “Broken” class. Many studies have highlighted the importance of these features and revealed higher rates of rupture in aneurysms associated with inflammatory markers. CRP, C3, and C4 levels may be indicative of systemic inflammation, which has been associated with vascular remodeling, thus aneurysm instability. Our results are in agreement with those of various studies [5,6,7].

Our study highlighted the role of circulating IgM relative to C3/C4 and IgG/IgA. In the RF feature importance plot, venous IgM (V.IgM) and, to a lesser extent, carotid IgM (C.IgM), ranked highly among biological predictors, together with C.C4/A.C4 and C.IgG. LIME corroborated this pattern: low IgM values (e.g., V.IgM ≤ 0.07–0.10, C.IgM ≤ 0.10–0.17) frequently contributed to “Broken” predictions, whereas higher IgM levels shifted several instances toward “Not-Broken”, while elevated C4 and IgG supported classification as “Broken”. By contrast, IgA appeared as a modest, context-dependent contributor rather than a primary discriminator. Boulieris et al. also observed that venous, parent-artery, and intra-aneurysmal IgM concentrations are lower in ruptured than in unruptured aneurysms, interpreting this primarily as increased wall deposition of IgM in the former [5]. Other structural features, such as HEIGHT and VERTI, had lower global importance and were generally larger in non-ruptured cases, and AR—despite its established clinical relevance—was overshadowed by inflammatory variables in this dataset. SEX had minimal impact on classification, with similar modeled risk in men and women. And as for the instance that was incorrectly classified, it had a low CRP level, which made it more consistent with the Not-Broken class. The misclassified instance’s moderate C3/C4 values made classification ambiguity worse. This contradiction in blood sample values and the wrong classification for this instance is strong evidence that CRP/C3/C4 are the most important features. The discrepancy that the misclassified instance had exceptionally high IgA and IgG levels, suggesting that the model undervalued their predictive power.

Another interesting finding is that neck size in the correlation matrix, RF feature importance plot, and LIME explainer are assigning a more prominent discriminative role to aneurysm neck size than to overall size or aspect ratio within an inflammatory context. Classical morphological studies, and subsequent reviews [1,56,57,58], emphasize dome size and aspect ratio as major predictors of rupture risk, with neck width receiving comparatively little independent attention. In our study, neck diameter exhibits one of the strongest correlations with aneurysm BROKEN status. Neck width is inversely related to inflammatory activity, implying that smaller neck aneurysms were correlated to higher A.CRP and V/C/A.C3 levels, which may suggest that tighter aneurysm necks are associated with greater inflammatory activity, thus instability. Also, neck width is the top-ranked geometric feature in the RF feature importance plot, immediately following the main inflammatory markers. Irregular shape also emerges as the most informative morphological predictor once inflammation is accounted for. It is moderately positively correlated with BROKEN and is the most important non-biochemical feature globally. LIME show that IR.SH regularly appears among the top local drivers of “Broken” predictions, and that specific small-neck ranges (e.g., 0.09 < NECK ≤ 0.19 or NECK ≤ 0.28) further increase rupture probability.

Beyond reproducing known associations between inflammation, complement activation, and rupture status, this study introduces an integrated, interpretable framework that jointly models multi-site inflammatory profiling and aneurysm morphology and explains each prediction at the patient level. Existing clinical and experimental literature typically addresses morphological risk factors (size, aspect ratio, irregular shape) separately from biochemical markers such as CRP, complement components, and immunoglobulins. Our findings instead delineate a composite high-risk phenotype—irregular shape, narrow neck, elevated C3 and CRP across vascular compartments, and reduced circulating IgM—that consistently characterizes aneurysms classified as ruptured in our cohort. This coupled geometry–inflammation profile is visible simultaneously in global RF feature importance, LIME, and empirical correlations, and is not explicitly described as a unified construct in prior work.

Relative to existing machine-learning studies that have focused mainly on morphological, radiomic, or hemodynamic descriptors, our model achieves strong discrimination while adding a rich biological layer. Most prior ML work on intracranial aneurysms has emphasized shape indices, wall shear stress, or imaging-derived radiomics, yielding powerful but predominantly geometry-driven models [8,9,10,11,14,15,16,17,18]. In contrast, our pipeline incorporates multi-site inflammatory biomarkers and complement activation alongside morphology, producing explanations that are explicitly bio-mechanistic (inflammation plus geometry) rather than purely geometric. Furthermore, by using a transparent ensemble (RF) and pairing global importance with LIME, we adhere to recommendations for interpretable medical ML, avoiding fully black-box architectures. Finally, the use of random oversampling, Optuna-based hyperparameter tuning, and performance metrics suited to class imbalance (ROC–AUC, AP), combined with out-of-fold estimates and a held-out test set, aligns with best practices for small, imbalanced clinical datasets and facilitates comparison with both statistical and ML literature.

Taken together, our results indicate that morphology is necessary but not sufficient for reliable rupture discrimination. Morphological features—particularly NECK and IR.SH—contribute consistently and meaningfully to the model, yet the largest shifts in rupture probability occur when adverse geometry co-occurs with elevated CRP/C3/C4. In many LIME profiles, irregular shape or narrow neck in the context of low inflammatory and complement activity produced only modest increases in predicted rupture risk, whereas the same geometry combined with high CRP and C3/C4 yielded substantial probability changes. This pattern provides a plausible explanation for why morphology-only models, even when sophisticated, can misclassify cases where the inflammatory state diverges from shape-based expectations. Biologically, the findings support a biomechano-inflammatory view of aneurysm instability: geometry shapes local hemodynamics, which modulate endothelial function and immune activation, and rupture propensity reflects the joint product of both processes rather than geometry alone. Consequently, integrated models that combine morphology with inflammatory and complement markers appear more appropriate for clinical decision support and for guiding future prospective validation efforts than morphology-only approaches.

Limitations: While our findings offer valuable insights, this study has limitations that should be noted. First, our sample size is limited to 35 patients. This is a direct reflection of the technical challenges and serious risks—such as arterial dissection or aneurysm rupture—involved in sampling blood directly from the carotid artery and the aneurysm sac. Given these constraints, our work is best characterized as a pilot or proof-of-concept study rather than a definitive large-scale analysis.

To make the most of the available data, we used robust validation methods like stratified five-fold cross-validation and oversampling. However, we recognize that these results might not fully represent broader or more diverse populations. Similarly, although we included sex as a variable in our models and found no clear patterns, the study is not large enough (underpowered) to rule out subtle sex-specific biological differences.

Finally, since this was a single-center observational study, we can only report associations rather than clear cause-and-effect. While we used tools like LIME to make our ML models more transparent, the models are still inherently tied to this specific dataset. Future research with larger, multi-center groups will be vital to confirm these early results.

## 5. Conclusions

Conclusions: This comparison highlights the advantages of machine learning approaches in incorporating nonlinear feature interactions, allowing for a more granular and individualized interpretation of rupture status. While statistical methods confirmed key biomarkers such as CRP and C3 as crucial indicators of aneurysm rupture, the LIME-based approach provided additional insights by integrating structural parameters, which were absent from the statistical analysis. The findings suggest that aneurysm morphology is not only relevant but may be mechanistically linked to inflammatory processes, offering new directions for further research.

Future studies could benefit from a hybrid approach that combines statistical validation with machine learning feature importance rankings, ensuring both interpretability and robustness in aneurysm rupture prediction models. The integration of morphological and inflammatory markers in predictive models may ultimately enhance early detection and clinical decision-making for patients at risk of aneurysm rupture.

## Figures and Tables

**Table 1:** Demographic data and aneurysm characteristics: M: male, F: female, ICA: internal carotid artery, C.T: carotid tip, P-comm: posterior communicating artery, A-comm: anterior communicating artery, MCA: middle cerebral artery, PCA: posterior cerebral artery, IA: intracranial aneurysm, (L, R left, right).

Patient	Age/Sex	Aneurysm Location	Size: Largest IA Diameter (mm)	Bleb	Irregular Shape	Aspect Ratio (mm)	Neck (mm)	Vertical to Neck (mm)	Event of Rupture
1	62/F	ACOM	8.0	NO	NO	2.0	4.0	8.0	NO
2	49/F	PERI	6.15	YES	YES	1.02	4.42	4.51	NO
3	49/F	L.MCA	8.32	YES	YES	1.94	2.58	5.0	YES
4	68/M	ACOM	4.91	YES	YES	1.57	2.76	4.32	YES
5	56/F	ACOM	7.83	YES	YES	1.38	2.8	3.85	YES
6	47/F	PCA	4.24	YES	YES	1.11	2.43	2.7	YES
7	55/M	ACOM	6.92	YES	YES	1.14	3.78	4.32	YES
8	68/M	R.PCOM	9.25	YES	YES	3.18	3.84	12.2	YES
9	39/F	L.PCOM	5.33	YES	NO	1.5	3.51	5.28	YES
10	46/F	L.ICA	7.48	YES	NO	1.22	4.18	5.08	NO
11	64/F	R.ICA	21.1	NO	YES	4.72	4.47	21.1	NO
12	54/M	ACOM	5.71	YES	YES	1.45	3.94	5.71	YES
13	75/F	L.MCA	4.75	YES	NO	1.45	3.27	4.75	YES
14	56/M	R.MCA	11.8	YES	YES	3.06	3.86	11.8	YES
15	65/F	ACOM	8.5	YES	NO	2.43	3.38	8.21	YES
16	67/F	L.PCOM	7.27	YES	YES	2.08	3.36	6.98	YES
17	44/F	L.ICA	3.47	YES	NO	1.39	2.49	3.47	NO
18	69/M	L.MCA	4.54	YES	YES	1.43	2.49	3.56	YES
19	37/F	ACOM	5.52	YES	NO	1.83	3.01	5.52	NO
20	70/F	ACOM	10.1	YES	YES	5.03	1.83	9.2	YES
21	73/F	ACOM	4.34	YES	YES	2.08	1.88	3.91	YES
22	36/M	L.C.T	8.04	YES	YES	2.36	3.41	8.04	NO
23	40/M	ACOM	6.77	YES	YES	0.75	2.76	2.06	YES
24	52/M	R.MCA	7.88	YES	YES	1.02	4.6	4.7	YES
25	52/M	L.MCA	2.68	YES	NO	1.02	2.63	2.68	NO
26	36/F	L.MCA	4.18	YES	NO	0.93	2.27	2.11	NO
27	78/M	ACOM	3.71	YES	NO	0.78	3.34	2.6	YES
28	64/F	R.MCA	7.18	YES	NO	1.17	3.61	4.23	NO
29	76/F	L.ICA	12.2	NO	NO	2.59	4.71	12.2	NO
30	74/M	ACOM	10.3	YES	NO	1.19	3.55	4.22	NO
31	53/F	R.PCOM	7.31	YES	YES	2.18	3.29	7.16	YES
32	62/F	L.PCOM	6.47	YES	NO	1.42	4.56	6.47	NO
33	60/M	ACOM	8.98	YES	YES	2.6	3.45	8.98	NO
34	59/F	L.ICA	25.0	YES	NO	2.43	10.3	25.0	YES
35	66/M	L.ICA	13.3	YES	NO	1.63	7.84	12.8	NO

**Table 2:** This matrix aids in analyzing model performance and identifying misclassifications.

Actual Class	Predicted Class →	
	Negative	Positive
Negative	TN	FP
Positive	FN	TP

**Table 3:** Means ± standard deviations across five-fold stratified CV on the oversampled training set.

Model	Accuracy	ROC AUC	F1	Sensitivity	Specificity
DT	0.690 ± 0.152	0.700 ± 0.151	0.579 ± 0.342	0.567 ± 0.365	0.833 ± 0.236
RF	0.690 ± 0.095	0.903 ± 0.095	0.658 ± 0.124	0.633 ± 0.247	0.767 ± 0.325
XGB	0.686 ± 0.157	0.833 ± 0.109	0.664 ± 0.175	0.633 ± 0.247	0.733 ± 0.279
AdaBoost	0.662 ± 0.107	0.786 ± 0.101	0.639 ± 0.129	0.633 ± 0.247	0.700 ± 0.298
GBM	0.595 ± 0.181	0.694 ± 0.234	0.488 ± 0.309	0.450 ± 0.310	0.767 ± 0.224

**Table 4:** The accuracy and ROC-AUC results of RF on train/test with its default settings and with stratified cross-validation.

Accuracy and ROC-AUC with Default Settings				
Method	CV Mean Accuracy	CV Std Accuracy	Train Accuracy	Test Accuracy
Stratified K-Fold	0.64	0.07	1.00	0.71
	CV Mean ROC-AUC	CV Std ROC-AUC	Train ROC-AUC	Test ROC-AUC
Stratified K-Fold	0.81	0.11	1.00	0.92

**Table 5:** The accuracy and ROC-AUC results of RF on train/test with optimized settings and with stratified cross-validation.

Accuracy and ROC-AUC with Optimized Settings				
Method	CV Mean Accuracy	CV Std Accuracy	Train Accuracy	Test Accuracy
Stratified K-Fold	0.85	0.09	0.91	0.86
	CV Mean ROC-AUC	CV Std ROC-AUC	Train ROC-AUC	Test ROC-AUC
Stratified K-Fold	0.98	0.07	0.97	0.92

**Table 6:** Test classification report.

Class	Precision	Recall	F1-Score	Support
false	0.75	1.00	0.86	3
true	1.00	0.75	0.86	4
accuracy	—	—	0.86	7
macro avg	0.88	0.88	0.86	7
weighted avg	0.89	0.86	0.86	7

**Table 7:** The seven instances of the test set and their probability of being in the Broken class.

Row	Index	True Label	Predicted Label	Probability Broken
0	10	False	False	0.380563
1	31	False	False	0.247458
2	21	False	False	0.445978
3	4	True	True	0.673814
4	3	True	True	0.696503
5	15	True	True	0.645672
6	11	True	False	0.419236

**Table 8:** Comparison of features among classes.

Feature (Sites)	Not-Broken	Broken	Strength/Notes
CRP (A.CRP, C.CRP, V.CRP)	~0 across sites	Non-zero at ≥1 site (often A/C≈0.02–0.06; V can be higher)	Strong separator.
C3/C4 (A.C3, C.C3, V.C3, A.C4, C.C4, V.C4)	Generally lower	Generally higher, especially V.C3/C4 and C.C3	Strong separator.
Immunoglobulins (V.IgA, V.IgG, V.IgM)	Lower to moderate	V.IgA/V.IgG tend higher; V.IgM varies	Moderate separator.
Immunoglobulins (A.IgA, A.IgM, A.IgG)	Mixed; often moderate	They can be lower (A.IgA/A.IgM) or high (A.IgG), depending on case	Weak–Moderate, direction not uniform.
Immunoglobulins (C.IgA, C.IgM, C.IgG)	Mixed	Mixed	Weak, no clean separation in this sample.
NECK	Larger (≈0.25–0.32)	Smaller (≈0.11–0.19)	Strong separator.
VERTI	Tends larger	Smaller	Moderate separator.
MD (max diameter)	Tends larger	Smaller	Moderate separator.
HEIGHT	Tends higher	Lower	Weak–Moderate.
AR (VERTI/NECK)	Variable; can be high (e.g., 0.93)	Usually low–moderate (≈0.15–0.31)	Weak alone; not decisive.
SEX	Appears in both	Appears in both	Not predictive here.
Irregular shape (IR.SH)	Appears in some Not-Broken	Present in all Broken in this set	Supportive but not specific;

## Data Availability

The data are available within the manuscript in Table 1.

## Abbreviations

The following abbreviations are used in this manuscript:

ACOM	Anterior communicating artery
ADASYN	Adaptive synthetic sampling
AP	Average precision
AR	Aspect ratio (verti/neck)
AUC	Area under the ROC curve
A.C3/A.C4	Aneurysm-sac complement C3/C4
A.CRP	Aneurysm-sac C-reactive protein
C3/C4	Complement component 3/4
C.C3/C.C4	Parent-artery (carotid) complement C3/C4
C.CRP	Parent-artery (carotid) C-reactive protein
CRP	C-reactive protein
CT	Computed tomography
CTA	Computed tomography angiography
CV	Cross-validation
DT	Decision tree
FN	False negative
FP	False positive
FPR	False-positive rate
GBM	Gradient boosting machine
IA	Intracranial aneurysm
ICA	Internal carotid artery
IgA/IgG/IgM	Immunoglobulin A/G/M
IR.SH	Irregular shape
L/R	Left/Right
L.C.T/C.T	(Left) carotid tip/Carotid tip
LOCI	Aneurysm location (feature)
MD	Maximum dimension (largest IA diameter)
MCA	Middle cerebral artery
ML	Machine learning
OOF	Out-of-fold (validation)
PCOM	Posterior communicating artery
PCA	Posterior cerebral artery
PR	Precision–recall
RF	Random forest
ROC	Receiver operating characteristic
ROC–AUC	Area under the ROC curve
ROS	Random oversampling
SAH	Subarachnoid hemorrhage
SHAP	SHapley Additive exPlanations
Skf-CV	Stratified k-fold cross-validation
SMOTE	Synthetic minority oversampling technique
TN	True negative
TP	True positive
TNR	True-negative rate (specificity)
TPR	True-positive rate (sensitivity)
UIA	Unruptured intracranial aneurysm
V.C3/V.C4	Peripheral-vein complement C3/C4
V.CRP	Peripheral-vein C-reactive protein
VERTI	Vertical distance neck→dome
WFNS	World Federation of Neurological Surgeons (scale)
XGB	Extreme gradient boosting (XGBoost)
